# Interactive Effects of Enalapril Administration and Novel HIIT Wheel-Bed Training in Aged Rats

**DOI:** 10.3389/fresc.2021.764686

**Published:** 2021-11-08

**Authors:** Youfeng Yang, Anisha Banerjee, Yi Sun, Christy S. Carter, Thomas W. Buford

**Affiliations:** ^1^Department of Medicine, University of Alabama at Birmingham, Birmingham, AL, United States; ^2^Center for Exercise Medicine, University of Alabama at Birmingham, Birmingham, AL, United States; ^3^Integrative Center for Aging Research, University of Alabama at Birmingham, Birmingham, AL, United States; ^4^Geriatric Research Education and Clinical Center, Birmingham VA Medical Center, Birmingham, AL, United States

**Keywords:** aging, forced exercise, renin-angiotensin system, multi-modal intervention, strength

## Abstract

**Introduction:** Growing research suggests that aerobic high-intensity interval training (HIIT) improves cardiovascular function and physical performance compared with moderate intensity continuous training (MICT). However relatively few animal models of HIIT are available to inform about the benefits of this exercise—particularly among older animals. In addition, there is little evidence for how HIIT training interacts with adjuvant pharmacological therapies known to enhance the impact of MCIT in older individuals such as Angiotensin Converting Enzyme (ACE) Inhibitors.

**Purpose:** The aim of the present study was to establish a HIIT protocol in aged rats based on forced running wheel-bed, and to subsequently (1) establish the feasibility of the HIIT protocol in a proof-of-concept study evaluating interactions between HIIT and (2) the result of combining HIIT + ACE inhibitor treatment using the ACE inhibitor enalapril.

**Methods:** Two groups of rats were used in this study. The feasibility of using wheel-bed for HIIT training was tested in group one (15- and 30-month-old male rats). In the second group, 37 24-month-old Fisher 344 x Brown Norway male rats were randomly divided into four subgroups: control, enalapril, HIIT training group, and HIIT training combined with enalapril administration. The training and administration lasted for 4 weeks. After the intervention, locomotor activity, exercise tolerance, and grip strength were tested.

**Results:** Our feasibility study suggested that middle-aged and aged rats were able to successfully complete the HIIT training. In our intervention study, HIIT training alone, regardless of adjuvant enalapril intervention, did raise treadmill exercise tolerance vs. the sedentary condition. Measures of healthspan were not negatively impacted by HIIT training.

**Conclusion:** The novel HIIT protocol based on forced running wheel-bed was successfully employed in aged rats. We conclude that future studies should compare the results and of multi-modal intervention strategies which include both HIIT and MICT in combination with adjuvant therapies such as enalapril to improve exercise tolerance and other global indices of healthspan.

## Introduction

High-intensity interval training (HIIT) is described as high-intensity exercise with intervals that are characterized by repeated sessions of high-intensity exercise period followed by rest or low-intensity exercise for recovery (1–3). For years, moderate-intensity continuous training (MICT) has been the most preferred exercise modality for improving body composition and cardiorespiratory fitness (4). Long-term devotion to this training method has however been low as many people stop exercising as a result of lack of time (4, 5). In comparison, HIIT is undoubtedly a time-efficient strategy to increase metabolism and enhance skeletal muscle capacity (3, 6). HIIT is also less monotonous, thereby increasing participation and adherence (7, 8). Furthermore, aerobically-based HIIT has been reported to extensively improve cardiorespiratory fitness—a strong determinant of morbidity and mortality (5)—and to stimulate an increase of serum brain-derived neurotrophic factor (BDNF) concentrations—a critical indicator for mental health (9). Additionally, aerobic HIIT leads to mitochondrial biogenesis and induces multiple factors involved in tissue regeneration (10, 11).

For decades, HIIT was primarily used in athletic practice for runners and swimmers (12). More recently, HIIT gained much attention for not only athletes but also as an effective exercise module in healthy adults in maintaining or enhancing body composition, body weight control and disease rehabilitation (5, 13). However, most of these studies were carried out with young adults. Fewer studies have been done on older adults, especially healthy older adults. Preliminary studies have demonstrated the low risk and feasibility of HIIT in older adults. High-intensity exercise training program has shown well-tolerance in individuals of 45–80 years old with Parkinson's disease (95% adherence) (14).

Using animal models in exercise research has long provided insights into the physiologic adaptations to exercise. Recently, animal models of HIIT have been developed, exclusively using mice as a model (15). However, as with any model, mice have certain disadvantages—particularly for studying aging. For instance, the advantage of using a rat model in comparison to a mice model lies in the ease of sample collection and the quantity of samples that can be collected (16). In exercise physiology and behavior studies, rats showed less data variability and are more repeatable (16). Therefore, the rat appears to be an important animal model in exercise-related research. Indeed, the rat is the preclinical model of choice for the Molecular Transducers of Activity Consortium (MoTrPAC), a national research consortium designed to discover and perform preliminary characterization of the range of molecular transducers (the “molecular map”) that underlie the effects of physical activity in humans. However, to our knowledge, the MoTrPAC group is currently not exploring the use of HIIT. The purpose of this study is to develop a rat HIIT protocol based on the forced running wheel-bed.

Presently, the treadmill is the most preferred apparatus for HIIT training in rats (17–19). However, a main limitation of treadmill training lies in that most treadmill systems utilize electrical shock or aversive air-puffs to motivate the animal to maintain activity (20). These stimuli are potentially confounding factors of exercise effects due to their stressful nature (21). Some animals refuse to run on the treadmill after a period of time and although not reported in the literature, personal communications indicate that there may be as much as a 20% dropout rate out due to the aversive nature of the protocol (22). Moreover, the flat treadmill surface does not necessarily imitate the natural environment (23).

Therefore, we explored the feasibility and effectiveness of a new apparatus for HIIT training. The forced running wheel-bed is designed as a forced exercise apparatus. Without the need for electric shock or other stimuli, the rat must continue to run as the wheel rotates. The circular track of the wheel-bed mimics the tunnel structures in the rat cave, with uphill and downhills, which are closer to their natural environment. We based the HIIT protocol on three pieces of evidence. First, our past studies (under revision for publication) have demonstrated that moderate intensity continuous training (MICT) treadmill exercise in rats (10 min/day—without the use of shock as a motivator) for as little as 4 weeks confers a benefit to exercise tolerance. Secondly, we have also previously demonstrated that very old male rats (28 months of age) (24) undergoing treadmill MICT for 25 min/day demonstrate improved exercise tolerance, although using shock in this paradigm was highly stressful to subjects. Thirdly, we utilized a protocol based in older frail mice demonstrating that a HIIT protocol is well-tolerated and improves physical performance (15). This mouse study was a 10 min protocol, like that used previously in our rats with MIT. However, in the mouse protocol, the time span was for 16 as opposed to 4 weeks. We did not use the speeds used in mice as our anchor. Instead, we first tested these older rats to see what they were capable of, hence our approach that our first experiment was necessary to help to establish these speeds. Thus, our protocol was based upon our own studies and the literature.

After pilot testing of the protocol, we also aimed to conduct a proof-of-concept/intervention study to demonstrate the utility of the protocol. For this study, we evaluated the interaction of HIIT with enalapril treatment, a commonly used pharmacologic agent with which our laboratory has extensive experience. Enalapril is an orally effective long-acting angiotensin-converting enzyme inhibitor, used to treat hypertension and chronic heart disease (25). Various clinical trials have shown that for patients with congestive heart failure, enalapril or related angiotensin-converting enzyme (ACE) inhibitor can reduce its mortality and morbidity (26, 27). Further studies have shown that enalapril has an important role in promoting the exercise capacity of patients with heart failure with reduced left ventricular ejection fraction (HFrEF) (28, 29). Compared with placebo, enalapril significantly increased the walking tolerance after treatment (28). In addition, genetic, epidemiological, and clinical studies in older adults demonstrate that multi-modal interventions of exercise/physical activity and ACE inhibitors improve physical function (30–34). However, none of these studies have investigated the impact of HIIT in combination with ACE inhibitor treatment. Therefore, we introduced this assessment into our animal model of aging.

Thus, the first aim of the present study was to determine if aged rats of typical used in aging research will participate in the new HIIT training protocol based on forced running wheel-bed. The second aim was to investigate the interactive effects of HIIT wheel-bed running and enalapril treatment on exercise tolerance in aged rats.

## Methods

### Animals

Two groups of rats were used in this study. The first group included four 30-month-old Brown Norway × Fischer 344 male (F1) male rats and six 15-month-old F344 male rats. These rats were available in our colony and were primarily used to test the feasibility of the wheel-bed device and the HIIT training protocols. It should be noted that these groups of animals were not tested in any subsequent tasks.

In the second group, 37 24-month-old Fisher 344 x Brown Norway rats were used. The age of these animals represents a translation to humans of ~60-years-old (35). The rats were randomly subdivided into the control group, enalapril administration group, HIIT training group, and HIIT training combined enalapril administration group. HIIT training was performed 5 times a week, 10 min per trial for 4 weeks. Enalapril doses used were administered subcutaneously by injection at 40 mg/kg body weight per day. This dose was based upon our previous studies (36). The training program and/or enalapril administration lasted for 4 weeks. Before and after the conclusion of the study, all animals were assessed using an exercise tolerance paradigm adjusted for older animals as well as grip strength and locomotor activity, both measures of relative healthspan as described previously by our group in the context of exercise and enalapril treatment experiments in older animals (24, 36). We assessed body weight weekly over the course of the experiment as an indicium of health in our animals.

For both experiments, animals were maintained on a 12 h light/dark cycle, with free access to a standard pellet rodent diet (18% kcal from fat, no sucrose, 3.1 kcal/g, diet 2018; Harlan Teklad, Madison, WI) and water *ad libitum*. All experiments described herein were pre-approved by the University of Alabama at Birmingham Animal Care Committee for the care and use of animals for scientific purposes.

### Wheel-Bed HIIT Training

**Figure 1:**
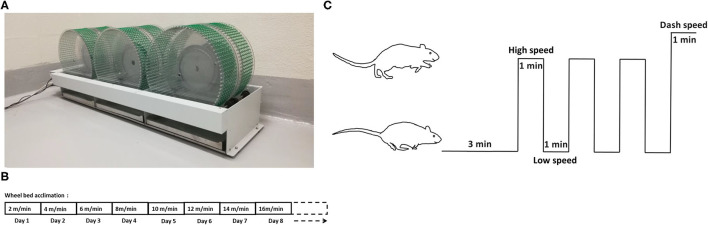
Wheel-bed and the HIIT acclimation and training protocol. **(A)** Wheel-bed used on aged rats. **(B)** Acclimation protocol in aged rats. **(C)** The HIIT training diagram based on wheel-bed.

The six-wheel rodent walking wheel-bed (Lafayette Instrument, Lafayette, IN) was utilized in our study (Figure 1A). Two weeks prior to testing, all rats were moved to the testing room for handling and acclimation to the wheel-bed and room. All rats were acclimatized to wheel-bed 10 min per day by walking with an increase of speed by 2 m/min each day, starting with 2 m/min until the maximum speed was reached. The maximum speed was usually reached after 5–7 days acclimation. The maximum running speed was recorded based on the performance defined by when the rat fell on the wheel three times within 60 s and could not gain back momentum on the wheel-bed (Figure 1B).

Following acclimation, the low speed, high speed, and dash speed for each rat was determined based on the calculation of 40, 80, and 100% of their individual maximum speed. HIIT habituated rats underwent 3-min warm-up period at low speed, followed by three intervals of 1-min at a constant high speed alternatively with 1-min at base speed, finishing with a final 1-min interval of dash speed (Figure 1C).

### Exercise Tolerance Test

After 4 weeks of intervention, an exercise tolerance test was performed. All rats were acclimatized to a five-lane rodent treadmill (Panlab/Harvard Apparatus) by walking at a speed of 8 cm/s, 10 min/day, for 3 days. Then all rats were running on the treadmill starting at 12 cm/s for 1 min, increased by 2 cm/s each min until 16 cm/s was achieved. Running time to exhaustion was recorded using a treadmill rather than the wheel-bed to compare across our previous studies (24).

### Forelimb Grip Strength Test and Locomotor Activity Test

Grip strength and locomotor activity are well-characterized measures known to translationally relate to health- and life-span across multiple species (37). We used these measures to identify if this potentially stressful HIIT protocol would impact healthspan.

The forelimb grip strength was determined using an automated grip strength meter (Columbus Instruments, Columbus Ohio). The rats were grasped by the tail and suspended above a grip ring. After about 3 s, the animal was gently lowered toward the grip ring and allowed to grasp the ring with its forepaws. The experimenter then quickly lowered the remainder of the animal's body to a horizontal position and tugged the animal's tail until its grasp of the ring was broken. The mean force in grams was determined with a computerized electronic pull strain gauge that is fitted directly to the grasping ring, and the resulting value normalized to body weight. Successful trials were defined as those in which the animal grasped the ring with both forepaws without jerking (38). The maximum measurement from three successful trials was taken as the outcome. For locomotor activity, animals were placed in activity chambers (42 × 56 cm) for 5-min sessions. Spontaneous activity was recorded with an overhead camera using the tracking system Ethovision XT (Noldus Information Technology Inc., Wageningen, the Netherlands). Total distance traveled (cm) in the arena was computed as the outcome measure.

### Statistics

For the feasibility experiment we ran two-tailed unpaired *t*-test to compare maximum speed achieved during HIIT training between the 15- and 30- month-old male animals to evaluate the impact of age.

For the intervention experiment, two-way analyses of variance (ANOVAs) with exercise (HIIT vs. sedentary) and treatment (enalapril vs. vehicle) are variables were used to establish main effects and interactions. Tukey's multiple comparisons test was then employed to confirm any specific interaction effects. All analyses were conducted using commercially available software (GraphPad Prism). Differences were considered statistically significant at *p* < 0.05.

## Results

### Forced Running Wheel-Bed on HIIT Training

**Figure 2:**
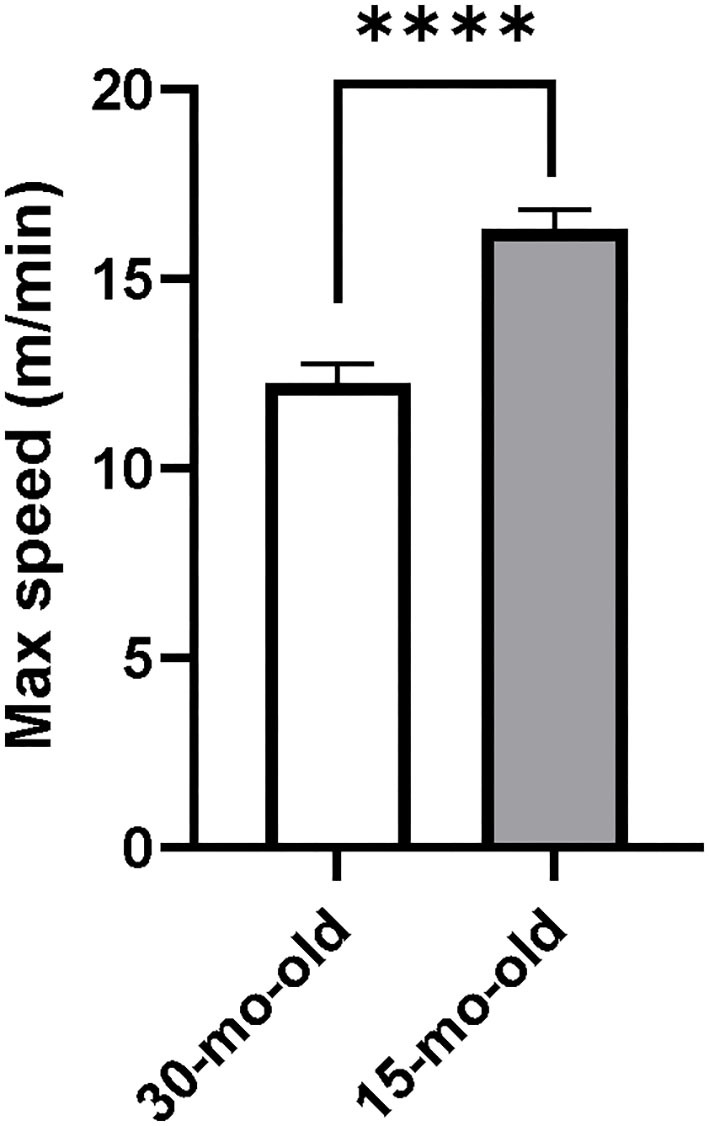
The dash speed of different aged rats in HIIT. ^****^p < 0.0001.

For the feasibility study, all 10 rats managed to complete the acclimation period and physically fit well on the forced running wheel-bed. The 30-month-old rats were able to adapt to HIIT training at a maximum speed of 12 m/min, while the 15-month-old rats were able to adapt to HIIT training at a maximum speed of 16 m/min (Figure 2). These speeds were statistically different from each other (*t* = 14.07, *p* < 0.01) indicating that middle-aged animals may train at higher speeds than the older animals. This finding helped us to determine the target maximal training speed for the older animals in our intervention study.

Rats randomized to the various exercise and treatment groups were gradually adapted to the wheel-bed device from low speed to high speed. Then, based on each individual rat's maximum speed, the actual speed for training was determined for each individual rat (Table 1). There were no observed main effects observed for exercise (*p* = 0.44) or treatment (*p* = 0.35) group assignment, nor any interactions (*p* = 0.06). All of animals were able to complete the training and the results below characterize the observed changes.

**Table 1:** The dash speed of rats in HIIT groups.

**ID**	**Low speed**	**High speed**	**Dash speed**
	**(m/min)**	**(m/min)**	**(m/min)**
HIIT 1	4	8	10
HIIT 2	5	10	12
HIIT 3	5	10	12
HIIT 4	5	10	12
HIIT 5	5	10	12
HIIT 7	5	10	12
HIIT 8	4	8	10
HIIT 9	5	10	12
HIIT 10	4	8	10
HIIT + Enalapril 1	5	10	12
HIIT + Enalapril 3	5	10	12
HIIT + Enalapril 4	5	10	12
HIIT + Enalapril 5	6	11	14
HIIT + Enalapril 6	5	10	12
HIIT + Enalapril 7	5	10	12
HIIT + Enalapril 8	5	10	12
HIIT + Enalapril 9	6	11	14

### Body Weight Changes

**Figure 3:**
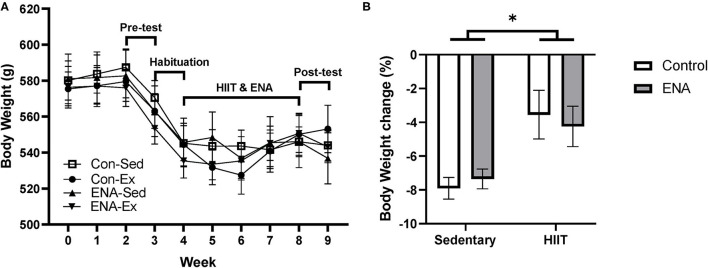
The body weight changes. **(A)** The time course of mean body weight changes during intervention and the timeline for training sessions and tests. **(B)** Body weight changes after the intervention. Con, control; Sed, sedentary; Ex, exercise; ENA, Enalapril. ^*^p < 0.05.

Due to the stressful nature of pre-behavioral testing and wheel-bed habituation, the average body weight of rats in each group dropped during pre-testing and habituation (Figure 3A). At the end of the intervention, % change in body weight was calculated for each animal and analyzed between groups. There were no effects of exercise (*p* = 0.87), treatment (*p* = 0.30) or any interactions (*p* = 0.91), although there was a tendency for animals in the HIIT condition to lose less weight (Figure 3B).

### Exercise Tolerance

**Figure 4:**
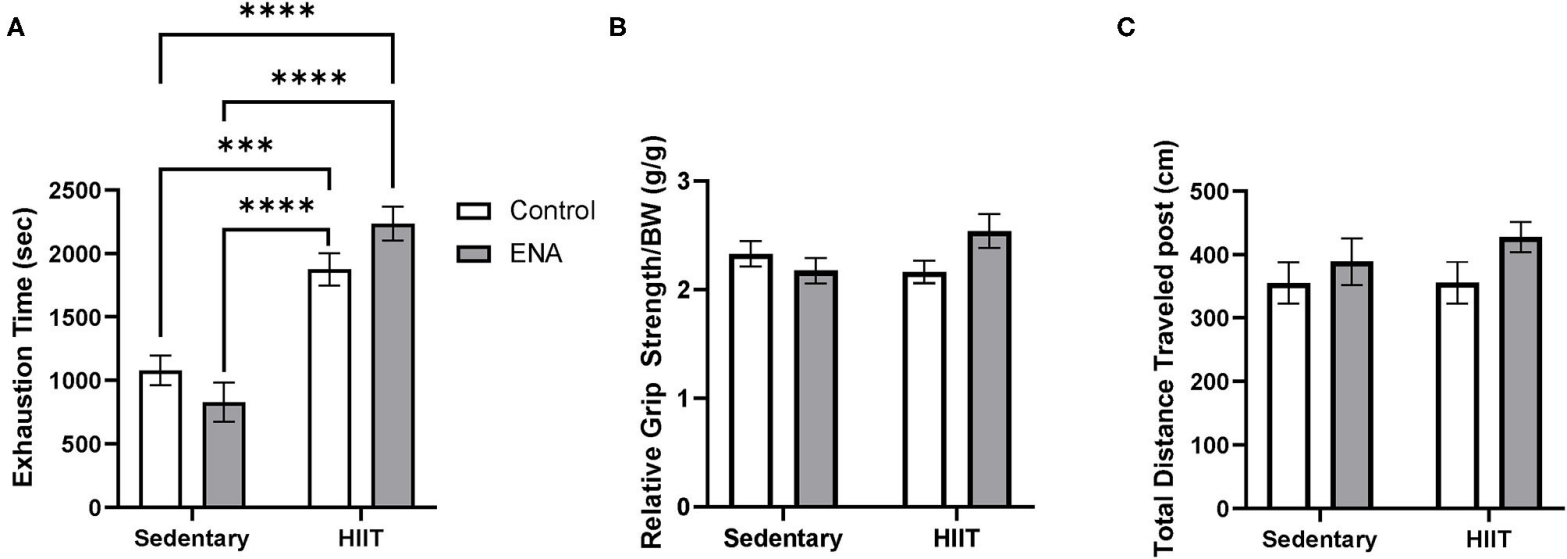
Exercise tolerance, relative grip strength, and locomotive assay. **(A**) Exercise tolerance after the intervention. **(B)** Grip strength by body weight after the intervention. **(C)** Total traveled distance in locomotor test. ^***^p < 0.001, ^****^p < 0.0001.

After four weeks of intervention, exercise tolerance was measured. The 2-way ANOVA revealed a main effect of exercise (*p* < 0.001) such that all exercised animals performed better than all sedentary animals (Figure 4A). There was also an interaction of exercise and treatment (*p* = 0.03). Tukey's *post-hoc* analysis, after correction for multiple comparisons, revealed no effect of treatment within exercise conditions.

### Locomotor Activity Test Forelimb Grip Strength

There were no differences between and pre- and post- intervention testing, therefore for simplicity we report results from the post-intervention testing.

There were no main effects were observed (*ps* > 0.05) for grip strength, but the interaction between treatment and exercise suggested there were significant differences amongst groups (*p* = 0.04) (Figure 4B). However, after running the Tukey's multiple comparisons, no interactive effects were observed (all comparisons *p* > 0.05). For locomotor activity, no main effects or interactions were observed for either factor (Figure 4C).

## Discussion

In the present study, we present a novel forced running wheel-bed HIIT training protocol that can be used in aged rats. This is the first rat study, to our knowledge, that adopted wheel-bed to this protocol.

Traditionally, treadmill is the most accepted apparatus for rats in HIIT training. The major advantage of the present protocol is that the wheel-bed provides a more natural environment for the rats as opposed to the treadmill and involves more skeletal muscles, both walking muscles and climbing muscles. Meanwhile, the treadmill only benefits walking muscles. Wheel running involves more muscle grip strength training. Indeed, all aged and middle-aged rats successfully accomplished the wheel-bed exercise. The new apparatus is feasible in terms of safety, technology, and operation.

The present HIIT protocol involving the wheel-bed has several advantages, namely,

- No electrical shock or other stimulations were used in this forced exercise system.
- It is easy to control both exercise intensity and duration with the wheel-bed.
- The acclimation process helped to mitigate the stressful effects of the new instrument and room environment, which diminishes the variability in training effects.
- With ascending and descending slopes and planes, the wheel-bed provides a more flexible environment to get more muscles involved in rat exercise.

Despite these advantages, certain limitations of the wheel-bed HIIT model were also observed. In our feasibility experiments, the maximum speed achieved in older animals was lower than what was achieved by the middle-aged rats. It is still unclear if this effect is strain or age-based given that two different strains were used and at different ages. Rats can tear and bleed their toenails during climbing and running. To avoid this, it is necessary to trim the toenails before the protocol.

In our intervention study, animals undergoing the HIIT protocol doubled their endurance time relative to their sedentary controls. While there was a trend for animals in the HITT + enalapril group to show better performance than HIIT alone, that difference was not significant. This this may be because the impact of HIIT was so profound that any further contribution of enalapril to the effect is masked.

A limitation of this study is that we used the treadmill to assess “exercise tolerance” as opposed to using the wheel-bed. Our thought was to use the treadmill to assess this outcome as this is the gold standard in preclinical studies. In fact, many labs use shock on the treadmill to motivate rats to run at high speeds. We have found that high speeds of training are not necessary to convey benefits to exercise tolerance, especially in older animals. And we have found that it is not necessary to use shock or air puffs to motivate rats to engage in “physical activity” at low speeds. However, some laboratories will continue to investigate high running speeds and will continue to suffer from attrition. Thus, if the wheel-bed, could we used as a forced exercise model, our study could provide an alternative approach.

HIIT is being used in human aging studies, (over 139 citations in PubMed) and most notably regarding age-related diseases, especially Parkinson's (39–41). However, to explore mechanisms, there is a need to use appropriate animal models and protocols to address these hypotheses. We do continue to question whether “Stressful” exercise paradigms in rats are translatable. Although in the current study, there was no indication that HIIT impaired general healthspan given that we observed no differences amongst groups on either grip strength or locomotor activity. This should be addressed in future studies with direct comparisons between “forced” and “voluntary” exercise. In addition, all researchers should consider if low level exercise, whether MICT or HIIT, could be differentially beneficial and whether adjuvant therapies could enhance these protocols. Our paper, to be sure, represents a first foray into this question and are hopeful that this information will be useful to other groups interested in implementing HIIT protocols in studies utilizing rats as a model.

To conclude, we present a new apparatus for HIIT protocol, using a forced running wheel-bed instead of the traditional treadmill. We also demonstrated that rats of different age and strain groups tolerated the exercise protocol very well. This rat HIIT model could benefit further studies exploring the pattern and mechanism of HIIT in the aged population in the context of aging and/or age-related disease.

## Data Availability Statement

The original contributions presented in the study are included in the article/supplementary material, further inquiries can be directed to the corresponding author/s.

## Ethics Statement

The animal study was reviewed and approved by Robert A. Kesterson, University of Alabama at Birmingham, IACUC Chair.

## Author Contributions

TB, CC, and YY: conceived and designed the experiments. YY, AB, YS, and CC: performed the experiments. YY, YS, and CC: analyzed the data. YY, CC, YS, AB, and TB: wrote the paper. All authors contributed to the article and approved the submitted version.

## Funding

This work was supported by the National Institutes of Health/National Institute on Aging (R01AG054538), the UAB Nathan Shock Center (P30AG50886), and the UAB Center for Exercise Medicine.

## Conflict of Interest

The authors declare that the research was conducted in the absence of any commercial or financial relationships that could be construed as a potential conflict of interest.

## Publisher's Note

All claims expressed in this article are solely those of the authors and do not necessarily represent those of their affiliated organizations, or those of the publisher, the editors and the reviewers. Any product that may be evaluated in this article, or claim that may be made by its manufacturer, is not guaranteed or endorsed by the publisher.
